# An Observational Study on C-Reactive Protein to Platelet Ratio in Neonatal Sepsis

**DOI:** 10.7759/cureus.62230

**Published:** 2024-06-12

**Authors:** Shreya Aggarwal, Avinash L Sangle, Mohd Saeed Siddiqui, Mohammad Haseeb, Madhuri B Engade

**Affiliations:** 1 Pediatrics, Mahatma Gandhi Mission Medical College and Hospital, Aurangabad, IND

**Keywords:** blood culture negative, positive blood culture, neonatal sepsis, c-reactive protein (crp) test, platelets in inflammation

## Abstract

Background: Neonatal sepsis is a serious medical condition affecting many individuals in the developing world. C-reactive protein (CRP) level in serum and platelet counts have been reported to have role in diagnosis of neonatal sepsis.

Objective: To evaluate the CRP to Platelet ratio (CPR) in relation to time and blood culture reports in neonatal sepsis patients from a tertiary care centre in the Marathwada region of Maharashtra.

Methods: The present observational study was conducted at the level III Neonatal Intensive Care Unit of a tertiary care centre in Aurangabad city of Marathwada region in Maharashtra from September 2022 to July 2023. The study included 120 neonates (delivered after completion of 28-42 weeks of gestation) with clinical/culture-positive sepsis. The newborns of seropositive mothers, neonates delivered in other hospitals, babies with congenital dysmorphic features, and babies requiring surgical procedures were excluded from the study.

Blood samples for complete blood count (CBC) and CRP were collected on days 1, 3 and 5. Blood cultures were sent on day 1 of illness. Repeated measures ANOVA was used to compare the parameters of CPR, CRP, and platelet count in blood culture-positive and blood culture-negative neonatal sepsis patients on days 1, 3 and 5.

Results: Blood culture was positive in 37 (30.8%) cases. A repeated measures ANOVA showed a significant overall difference in the CPR across days 1, 3, and 5 (p = 0.006). The CPR was significantly higher in culture-positive neonates compared to culture-negative neonates (p = 0.042).

Conclusion: Higher CPR in blood culture-positive neonates compared to blood culture-negative neonates supports the role of CPR in the diagnosis and management of neonatal sepsis.

## Introduction

Neonatal sepsis is a life-threatening clinical condition encountered by clinicians worldwide and a significant cause of neonatal morbidity and mortality, with disproportionate prevalence in low-income and middle-income countries [1-3]. A recent systematic review estimated that the population-level burden of neonatal sepsis was 22 per thousand live births, with 11-19% mortality [4].

Blood culture is the gold standard for diagnosing neonatal sepsis. However, the challenges associated with blood culture are extended time requirements, collection of an adequate blood volume, and prior empirical antimicrobial treatment [5-6]. The C-reactive protein (CRP) level has gained importance as a laboratory test in managing neonatal sepsis, as it has been found to help in diagnostic and prognostic evaluation. However, CRP level has yet to be efficiently validated as a screening biomarker [7-13]. The significance of platelets in the pathogenesis of sepsis has been demonstrated, and low platelet counts have been observed in severe sepsis [14-15].

The CRP to platelet ratio (CPR) is currently being evaluated for its role in neonatal sepsis. Li et al. reported that higher CPR was associated with the presence and severity of neonatal sepsis [16]. The present study evaluated CPR in neonatal sepsis patients from a tertiary care center in the Marathwada region of Maharashtra.

## Materials and methods

The present observational study was conducted from September 2022 to July 2023 at the level III Neonatal Intensive Care Unit of a tertiary care centre in Aurangabad city of the Marathwada region in Maharashtra. The Institutional Ethics Committee at Mahatma Gandhi Mission Medical College and Hospital, Aurangabad approved the study protocol (approval number MGM/Pharmac/ECRHS/2022/134).

Neonates (delivered after completion of 28-42 weeks of gestation) with clinical/culture-positive sepsis were included in the study. Newborns of seropositive mothers, neonates delivered in other hospitals, babies with congenital dysmorphic features, and babies requiring surgical procedures were excluded from the study. Blood samples for complete blood count (by 5-part cell counter) and CRP (by quantitative immunoturbidimetric method) were collected on days 1, 3, and 5. Blood cultures (BacT/Alert method) were obtained on day 1 of the illness.

Neonatal sepsis was defined as described by Aydemir et al., who used the European Medicines Agency report on the Expert Meeting on Neonatal and Paediatric Sepsis [17-18]. Neonates with at least two clinical findings and at least two laboratory findings regarding clinical, hemodynamic, tissue perfusion, or inflammatory variables mentioned below in the presence of a result of suspected or proven infection (positive culture), and for whom there was no other reason to explain these findings other than infection, were diagnosed with sepsis.

Clinical findings of neonatal sepsis include (1) Modified body temperature: (i) Core temperature greater than 38.5 °C or less than 36 °C and/or (ii) Temperature instability, (2) Cardiovascular Instability: (i) Bradycardia, (ii) Tachycardia, (iii) Rhythm instability, (iv) Reduced urinary output (less than 1 ml/kg/h), (v) Hypotension, (vi) Mottled skin, (vii) Impaired peripheral perfusion, (3) Skin and subcutaneous lesions: (i) Petechial rash, (ii) Sclerema, (4) Respiratory instability: (i) Apnoea or (ii) Tacypnoea or (iii) Requirement for ventilation support, (5) Gastrointestinal: (i) Feeding intolerance (ii) Poor sucking (iii) Abdominal distention, (6) Nonspecific: (i) Irritability (ii) Lethargy (iii) Hypotonia.

Laboratory findings of neonatal sepsis include (1) Leucocyte count: (i) < 4000/mm^3^ or (ii) > 20,000/mm^3^, (2) Immature to total neutrophil ratio: ≥0.2, (3) Platelet count < 100,000 /mm^3^, (4) C-reactive protein level > 15 mg/L or procalcitonin level ≥ 2 ng/mL, (5) Glucose intolerance confirmed at least 2 times: (i) Hyperglycaemia (blood glucose > 180 mg/dL or 10 mmol/L) or (ii) Hypoglycaemia (glycaemia< 45 mg/dL or 2.5 mmol/L), (6) Metabolic acidosis: (i) Base excess (BE) < − 10 mEq/L or (ii) Serum lactate > 2 mmol/L.

Repeated measures ANOVA was used to compare the parameters of CPR, CRP, and platelet count in culture-positive and culture-negative neonates on days 1, 3, and 5.

## Results

Of the 120 subjects, 73 (60.8%) were males and 47(39.2%) were females. Blood cultures were positive in 37 patients(30.8%). The mean weight in neonates with positive blood cultures was 2129 ± 612 g, whereas in neonates with negative blood cultures, it was 2446 ± 759 g.

Table 1 shows the mean values of CRP on days 1, 3, and 5 in neonates with positive blood culture and neonates with negative blood culture results. A repeated measures ANOVA revealed that CRP levels varied across different time points (p<.001). However, the interaction between time and culture report was not statistically significant (p=0.194). On day 1, CRP levels were significantly lower than those on days 3 (p = 0.011) and 5 (p < .001). On day 5, CRP levels were significantly higher than those on day 3 (p = 0.044). 

**Table 1:** C-reactive protein values on days 1, 3 and 5 in neonates with blood culture positive and culture negative SD: Standard deviation

Serum C-reactive protein (mg/L)	Culture negative [Mean(SD)]	Culture positive [Mean(SD)]
Day 1	15.87 (19.757)	9.076 (13.962)
Day 3	19.842 (21.077)	20.771 (34.76)
Day 5	28.121 (32.678)	37.32 (43.756)

Table 2 shows the platelet values on days 1, 3, and 5 in neonates with positive and negative blood culture results. Repeated measures ANOVA revealed no significant overall difference in platelet count across days 1, 3, and 5 (p = 0.949). In addition, the platelet count did not significantly differ between the culture-positive and culture-negative neonates (p = 0.497).

**Table 2:** Platelet values on days 1, 3 and 5 in neonates with blood culture positive and culture negative SD: Standard deviation

Platelets (10^5^/mm^3^)	Culture negative [Mean(SD)]	Culture positive [Mean(SD)]
Day 1	1.773 (0.731)	2.138 (1.486)
Day 3	1.635 (1.018)	2.015 (1.747)
Day 5	1.791 (1.224)	1.604 (1.423)

Table 3 shows the CPR values on days 1, 3, and 5 in neonates with positive blood culture results and neonates with negative blood cultures. A repeated measures ANOVA showed a significant overall difference in CPR across days 1, 3, and 5 (p = 0.006). The CPR significantly increased from day 1 to day 3 (p = 0.015) and from day 1 to day 5 (p = 0.005). The CPR was significantly higher in culture-positive neonates than in culture-negative neonates (p = 0.042).

**Table 3:** C-reactive protein to platelet ratio values on days 1, 3 and 5 in neonates with blood culture positive and culture negative SD: Standard deviation

C-reactive protein to platelet ratio	Culture negative [Mean(SD)]	Culture positive [Mean(SD)]
Day 1	14.461 (26.683)	5.619 (9.796)
Day 3	22.031 (35.534)	67.238 (207.482)
Day 5	23.227 (47.424)	103.198 (320.474)

## Discussion

Our study adds the distinctive trend of CPR across days in neonates with culture-positive and negative sepsis. The CPR was significantly higher in culture-positive neonates compared to culture-negative neonates. The study results support the role of CPR assessment in diagnosing and managing neonatal sepsis. The interrelationship of serum CRP, platelet counts, and other haematological parameters has been an area of interest in recent research studies in neonatal sepsis diagnosis and management. Mondal et al.'s study in Kolkata, India, observed a high sensitivity of serum CRP for neonatal sepsis. However, the specificity was low, and a combination of serum CRP with other haematological parameters was suggested for better assessment [19]. Jethani et al. utilised immature to total neutrophil ratio, absolute neutrophil count, and serum CRP. They reported that these parameters were helpful in the early diagnosis of neonatal sepsis [20].

Platelets play an essential role in thrombosis and immune responses. The interaction of platelets with white blood cells via adhesion molecules and immune modulators plays a vital role in inflammation. Also, platelets may contribute to disseminated intravascular coagulation and micro-thrombosis, which further lead to complications of sepsis and adversely impact organ function. Tang et al.'s study reported that serum platelet count decrease was a predictive marker in early-onset bacterial sepsis in neonates [21]. Khadka et al.'s study has also highlighted the significance of serum CRP and platelet count as markers for neonatal sepsis, especially in low-income countries [22]. Clinical research studies have demonstrated that lower platelet count is a biomarker for the severity of sepsis [16,23-25]. Thus, there is evidence of the role of CRP and platelet count in the diagnostic and prognostic evaluation of sepsis. The ratio of CRP to platelet count is a better tool for assessing neonatal sepsis as it accounts for sepsis patients' inflammatory and coagulation status. Li et al. reported that higher CPR could be an independent predictor of the presence and severity of neonatal sepsis [16].

Study limitations

The limitations include a small sample size, a single-centre study that included only babies born in our hospital, and the inability to distinguish between early and late sepsis. Despite these limitations, the results of our study are promising. They favour the utility of CPR in diagnosing neonatal sepsis, suggesting a potential avenue for improving neonatal care. Future studies with larger samples, multiple centres, and more comprehensive parameters will undoubtedly shed more light on the significance of CPR in managing neonatal sepsis.

## Conclusions

Based on the study results, it can be concluded that there is an increasing trend in the CRP-to-platelet ratio in neonates with clinical signs of sepsis. A higher CRP-to-platelet ratio in culture-positive neonates than in culture-negative neonates supports the role of the CRP-to-platelet ratio in the diagnosis and management of neonatal sepsis. Analysis of the CRP-to-platelet ratio can aid in the diagnosis of neonatal sepsis.
